# Left ventricular remodelling and hypertrophy in patients with aortic stenosis: insights from cardiac magnetic resonance imaging

**DOI:** 10.1186/1532-429X-13-S1-O37

**Published:** 2011-02-02

**Authors:** Marc R Dweck, Sanjiv Joshi, Tim Murigu, Ankur Gulati, Isabelle Roussin, Andrew Jabbour, Winston Banya, Alicia Maceira, Francisco Alpendurada, Nicholas Boon, Raad Mohiaddin, David Newby, Dudley Pennel, Sanjay Prasad

**Affiliations:** 1 Royal Brompton Hospital, London, UK; 2 University of Edinburgh, Edinburgh, UK

## Introduction

The response of the left ventricular myocardium to aortic stenosis has been incompletely characterised. Here, we sought to investigate the correlation between the severity of aortic stenosis and the hypertrophic response and to define the patterns of remodelling and hypertrophy with CMR.

## Methods

Consecutive patients with moderate or severe AS (aortic valve area <1.5cm^2^), normal coronary arteries and no other significant valve lesion or cardiomyopathy were scanned by 1.5T magnetic resonance and compared with contemporary age- and sex-matched healthy, control subjects. The extent and patterns of hypertrophy were assessed from volumetric cine images. Valve severity was assessed by planimetry and velocity mapping. Asymmetric forms of remodelling and hypertrophy were defined as having a septal-to-lateral wall thickness ratio >1.5.

## Results

Ninety-one patients (61±21 years;63% male) with aortic stenosis (AVA 0.93±0.32cm^2^) underwent CMR. The degree of hypertrophy was unrelated to aortic stenosis severity (p=0.53) and there was a wide variation in LV structure comprising normal ventricular geometry (n=11), concentric remodelling (n=11), asymmetric remodelling (n=11), concentric hypertrophy (n=33), asymmetric hypertrophy(n=15) and eccentric hypertrophy (n=10).

**Figure 1:**
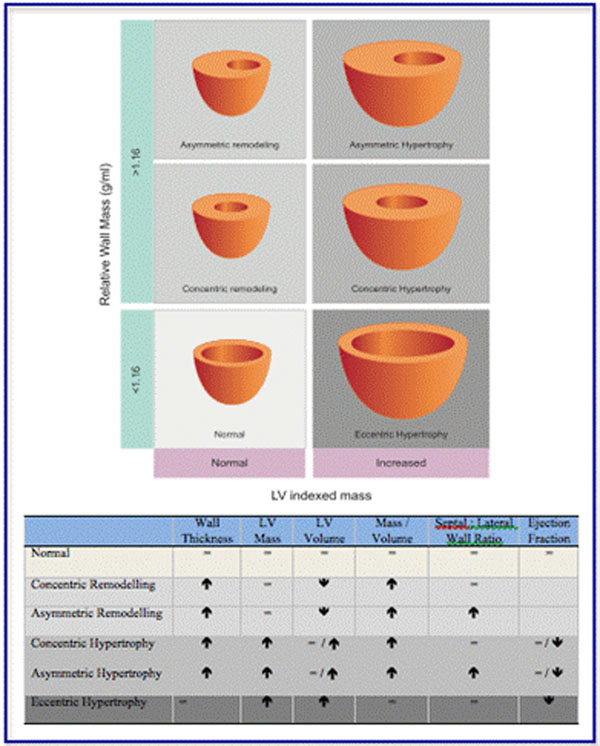
Cardiac magnetic resonance imaging definitions of LV hypertrophy and remodelling

**Figure 2:**
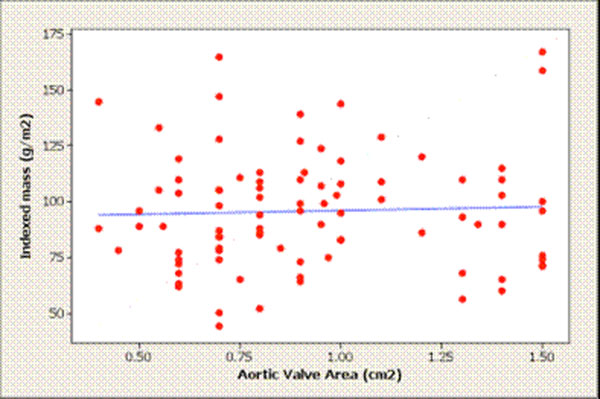
Influence of the Aortic Valve Area on the indexed LV mass. Pearson correlation: R=0.068; P= 0.530.

**Figure 3:**
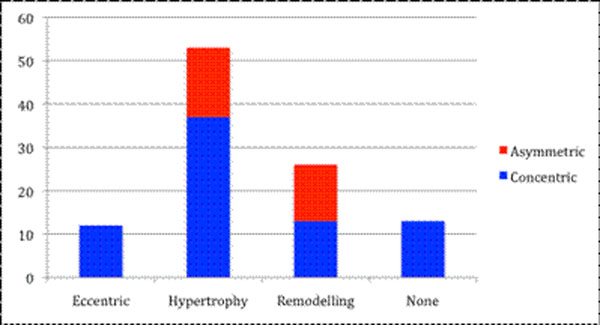
Prevalence of the different patterns of remodelling and hypertrophy in aortic stenosis (% of total cohort)

Asymmetric forms of remodelling and hypertrophy were observed in 29% of the cohort with considerable overlap in appearances (wall thickness 17±2mm) with hypertrophic cardiomyopathy. Figures 1, 2, 3, Tables 1, 2.

**Table 1:** Univariate predictors of increased left ventricular mass

Variable	Mean difference in Indexed Mass	Confidence Intervals	P value
Age > 66 years	7.51	-3.37- 18.39	0.17
Male sex	13.76	2.78 - 24.74	0.02
Moderate aortic stenosis	3.94	-7.62 - 15.50	0.50
Bicuspid valve	-7.27	-18.4 - 3.86	0.20
Hypertension	9.94	-1.05 - 20.93	0.08
Diabetes mellitus	11.91	-3.59 - 27.41	0.13
ACE Inhibitor / ARB	11.16	-0.99 - 23.31	0.07
β-Blocker	3.18	-11.24 - 17.60	0.66

**Table 2:** Baseline data of aortic stenosis patients with different forms of remodelling and hypertrophy

	Eccentric Group	Hypertrophy	Remodelling	Normal Ventricle
Number	10	48	22	11
Male sex (%)	60	65	68	45
Age (years)	69±18	62±18	62±18	52±26
Asymmetric Pattern (%)	-	31	50	-
Indexed LVEDV (ml/m2)	126±34	78±21	56±11	76±9
Indexed Mass (g/m2)	106±18	111±22	76±9	63±11
MASS/ VOLUME (g/mL)	0.88±0.19	1.50±0.31	1.40±0.31	0.84±0.16
Ejection Fraction (%)	45±16	69±13	76±12	73±5
Aortic valve area (cm2)	0.80±0.16	0.94±0.32	1.00±0.38	0.85±0.30

## Conclusions

We describe six different patterns of LV anatomic adaption to AS and wide variation in the degree of hypertrophy, which occurred independently of the severity of valve narrowing. These findings are likely to impact on imaging interpretation of aortic stenosis severity and may predict operative risk and the potential for reverse remodelling post-intervention.

